# Editorial: Circadian rhythms and exercise in cardiometabolic health

**DOI:** 10.3389/fendo.2023.1180851

**Published:** 2023-03-21

**Authors:** Milena Schönke, Karyn A. Esser, Brendan M. Gabriel

**Affiliations:** ^1^ Department of Medicine, Division of Endocrinology, Leiden University Medical Center, Leiden, Netherlands; ^2^ Einthoven Laboratory for Experimental Vascular Medicine, Leiden University Medical Center, Leiden, Netherlands; ^3^ Department of Physiology and Aging, College of Medicine, University of Florida, Gainesville, FL, United States; ^4^ Aberdeen Cardiovascular & Diabetes Centre, The Rowett Institute, University of Aberdeen, Aberdeen, United Kingdom

**Keywords:** circadian rhythms, physical activity, exercise, skeletal muscle, metabolic health

## Introduction

Regular exercise and physical activity are a cornerstone of the prevention and treatment of widespread cardiometabolic diseases such as obesity, type 2 diabetes (T2D), and cardiovascular diseases. On a cellular and molecular level, virtually all processes dysregulated in disease and involved in the response and adaptation of the body to exercise training are downstream of tissue biological clocks and their alignment within the body (1). This circadian regulation that allows our bodies to adapt to the changes of day and night in the span of 24 hours modulates energy metabolism, cell repair and renewal, and even immunological processes (2). Therefore, it is clear that time-of-day is an important factor in maximizing the health benefits of exercise and physical activity for disease prevention and treatment.

Several preclinical studies have shown that exercise can shift the muscle clock and both preclinical and clinical studies have demonstrated a time-of-day dependent effect of exercise on gene expression and energy metabolism (3–6). However, it is currently unclear when the “best” time to train is to achieve the greatest positive impact on metabolic health due to many gaps in our understanding of exercise, circadian clocks and metabolic health. For example, the complexity of how tissue clocks manage and integrate intrinsic neuroendocrine time cues associated with a person’s chronotype with extrinsic environmental cues such as light exposure, sleep, timing of food intake and timing of exercise is poorly understood in both health and disease. This Research Topic reviews current advancements that underline the value of timed exercise and physical activity as therapeutic interventions for cardiometabolic diseases in the era of chronomedicine.

## Exercise timing in cardiometabolic health

Hypertension, one of the components of the metabolic syndrome and strongly associated with cardiac deaths, responds to exercise training (7, 8). As the blood pressure naturally spikes in the morning, Imamura et al. investigated the association between blood pressure and habitual physical activity timing throughout the day in a cohort of 2343 Japanese men. Interestingly, individuals who performed vigorous exercise in the evening between 6 and 9 PM had the lowest systolic and diastolic blood pressure while individuals who went on walks in the very early morning between 3 and 6 AM had significantly higher blood pressure. This data matches the report by Arciero et al. who observed in an intervention study that evening exercise training more effectively lowered the blood pressure of exercise-trained men. Surprisingly however, in women, morning exercise achieved a greater reduction in blood pressure and abdominal fat mass while evening training on the other hand was found to increase muscular performance. This suggest that the ideal timing of physical activity may not be a “one size fits all” matter as health promoting effects and performance improvement may not always be synchronized since they are influenced by a number of factors such as age, sex or metabolic diseases. Improvements to metabolic homeostasis in individuals with obesity or T2D have previously been reported to be greater with evening exercise in short-term studies (9, 10). In a new randomized controlled trial with individuals with T2D, Liu et al. aim to further assess the effect of four months of combined exercise training paired with a controlled diet on fat mass, muscle mass and the role of the microbiota-gut-brain axis in exercise-induced metabolic benefits. This work will aid the optimization of exercise recommendations for individuals with T2D and obesity.

Over the past decades, the cellular and molecular effects of physical activity on skeletal muscle and the rest of the body have been investigated thoroughly and it has become evident that nearly every known aspect of this physiological response is intertwined with circadian rhythmicity (11). Bennet and Sato reviewed the current understanding of how exercise as an external cue is integrated with these internal circadian physiological processes to maintain metabolic homeostasis, identifying the opportunity to further research the role of “exerkines” (exercise-induced signaling molecules) and transcription factors that may constitute pharmacological targets.

## Exercise as a tissue rhythm keeper

Investigating the transcriptional impact of exercise training in a state of circadian disturbance, Lin et al. identified that high intensity interval training was able to offset the negative consequences of sleep impairment. In particular, exercise training reversed the induction of a pro-inflammatory transcriptional profile induced by five nights of sleep restriction in healthy men. This work highlights the importance of robust circadian rhythms for the maintenance of cardiometabolic health and points towards the capacity of exercise to re-align these rhythms should they be disturbed through sleep loss, jetlag, shift work or light exposure at night (12). Zooming in on skeletal muscle, Mansingh and Handschin reviewed the intracellular interaction of energy sensors such as AMPK or SIRT1 with the core clock machinery during muscle contractions, highlighting that the skeletal muscle clock is less sensitive to circadian perturbations like nighttime eating than, for instance, the highly susceptible liver clock. To investigate the efficacy and combinability of several phase shifting stimuli that can improve the body’s adaptation to a new light-dark rhythm, Youngstedt et al. designed a study exposing individuals to bright light or to bright light in combination with exercise and melatonin following an artificial, lab-induced 8 hour jetlag. The authors hypothesize that the combination of these known “Zeitgebers” will achieve quicker synchronization of the internal and external rhythm and will accordingly be the least detrimental to sleep, mood, performance, and sleepiness which is a common issue for shift workers that constitute 20% of the workforce.

## Perspective

**Figure 1:**
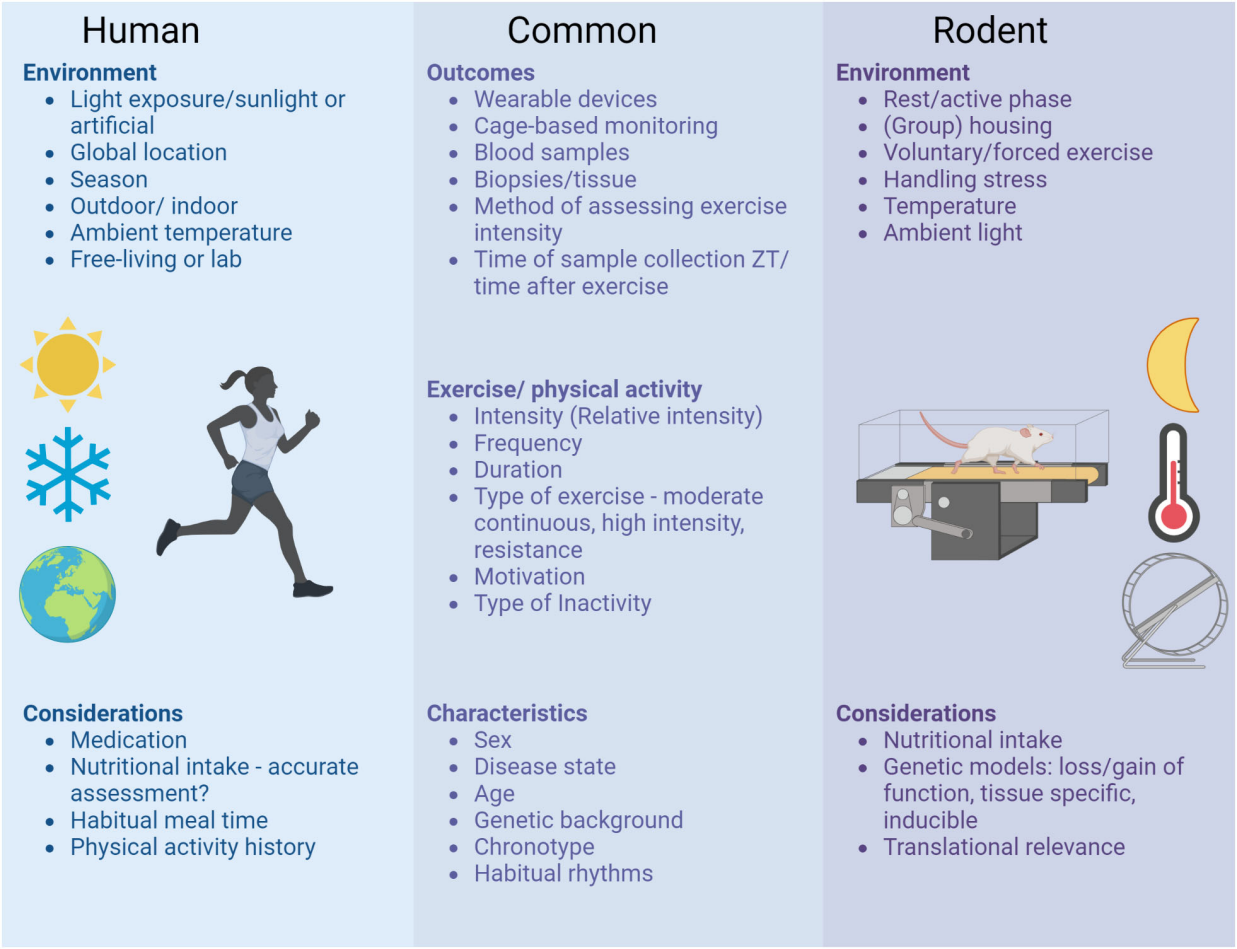
Considerations for study design and reporting of circadian exercise studies. To ensure the reproducibility and comparability of preclinical and clinical circadian exercise studies in the future, several aspects should be taken into account when planning, executing and reporting studies. These considerations range from unique aspects of clinical studies in free-living individuals to highly controlled lab environments of rodent studies and comprise common aspects relevant for all circadian exercise studies.

Optimizing lifestyle recommendations to include the “best” time to exercise bears great potential to maximize the efficacy of exercise training in health management - alone or in combination with drug treatments. To ensure the reproducibility and comparability of pre-clinical and clinical circadian exercise studies in the future, several considerations relevant for study design and reporting are summarized in **Figure 1**. As large-scale human studies are still limited at this point, the current recommendation remains: Any exercise at any time in the day is (likely) better than no exercise but it might be best to keep your time of exercise consistent.

## Author contributions

MS wrote the manuscript, KE and BG reviewed and edited the manuscript, BG created the figure. All authors contributed to the article and approved the submitted version.
